# Trans-Equatorial Migration Routes, Staging Sites and Wintering Areas of a High-Arctic Avian Predator: The Long-tailed Skua (*Stercorarius longicaudus*)

**DOI:** 10.1371/journal.pone.0064614

**Published:** 2013-05-21

**Authors:** Olivier Gilg, Børge Moe, Sveinn Are Hanssen, Niels Martin Schmidt, Benoît Sittler, Jannik Hansen, Jeroen Reneerkens, Brigitte Sabard, Olivier Chastel, Jérôme Moreau, Richard A. Phillips, Thomas Oudman, Elisabeth M. Biersma, Anette A. Fenstad, Johannes Lang, Loïc Bollache

**Affiliations:** 1 Laboratoire Biogéosciences, UMR CNRS 5561, Université de Bourgogne, Dijon, France; 2 Groupe de Recherche en Ecologie Arctique, Francheville, France; 3 Norwegian Institute for Nature Research (NINA), Tromsø, Norway; 4 Department of Bioscience, Aarhus University, Roskilde, Denmark; 5 Arctic Research Centre, Aarhus University, Aarhus C, Denmark; 6 Institut für Landespflege, University of Freiburg, Freiburg, Germany; 7 University of Groningen, Animal Ecology Group, Groningen, The Netherlands; 8 Centre d′Etudes Biologiques de Chizé, CNRS, Chizé, France; 9 British Antarctic Survey, Natural Environment Research Council, High Cross, Madingley Road, Cambridge, United Kingdom; 10 Arctic Centre, University of Groningen, Groningen, The Netherlands; 11 Royal Netherlands Institute for Sea Research, Den Burg, Texel, The Netherlands; 12 Department of Biology, Norwegian University of Science and Technology, Trondheim, Norway; 13 Institute of Animal Ecology and Nature Education, Gonterskirchen, Germany; Institut Pluridisciplinaire Hubert Curien, France

## Abstract

The Long-tailed Skua, a small (<300 g) Arctic-breeding predator and seabird, is a functionally very important component of the Arctic vertebrate communities in summer, but little is known about its migration and winter distribution. We used light-level geolocators to track the annual movements of eight adult birds breeding in north-east Greenland (n = 3) and Svalbard (n = 5). All birds wintered in the Southern Hemisphere (mean arrival-departure dates on wintering grounds: 24 October-21 March): five along the south-west coast of Africa (0–40°S, 0–15°E), in the productive Benguela upwelling, and three further south (30–40°S, 0–50°E), in an area extending into the south-west Indian Ocean. Different migratory routes and rates of travel were documented during post-breeding (345 km d^−1^ in late August-early September) and spring migrations (235 km d^−1^ in late April) when most birds used a more westerly flyway. Among the different staging areas, a large region off the Grand Banks of Newfoundland appears to be the most important. It was used in autumn by all but one of the tracked birds (from a few days to three weeks) and in spring by five out of eight birds (from one to more than six weeks). Two other staging sites, off the Iberian coast and near the Azores, were used by two birds in spring for five to six weeks. Over one year, individuals travelled between 43,900 and 54,200 km (36,600–45,700 when excluding staging periods) and went as far as 10,500–13,700 km (mean 12,800 km) from their breeding sites. This study has revealed important marine areas in both the south and north Atlantic Ocean. Sustainable management of these ocean basins will benefit Long-tailed Skuas as well as other trans-equatorial migrants from the Arctic.

## Introduction

Seabirds are among the most threatened organisms on Earth and their status has rapidly deteriorated in recent decades [1]. In addition to well-known threats faced at sea (competition with, and incidental mortality in fisheries, pollution, etc), there is growing concern about direct and indirect impacts of climate change [2]. Actions needed to mitigate these threats include site protection, especially of the most important marine areas (i.e. the key feeding and aggregation sites) within an international network of marine protected areas [1]. However, our capacity to design and implement such a network, requires much improved knowledge of the spatiotemporal distribution of seabirds, information on which is still very poor for many of the ca. 350 known species.

The Long-tailed Skua *Stercorarius longicaudus* has one of the more extreme life-styles of any Arctic bird. It functionally links different seabird species (kleptoparasitizing several small gulls and terns), ecosystems (being strongly dependent on terrestrial resources in summer and marine prey during the rest of the year) and even biomes (spending the summer in the Arctic and the winter in the Southern Hemisphere) [3]–[4]. Breeding as far north as any bird in Arctic tundra and polar desert ecosystems [5], its main summer diet usually consists of lemmings which have cyclic population dynamics that strongly impact the breeding success of the skuas, and are in turn influenced by this predation [6]–[8]. When lemmings are scarce or absent, as in Svalbard [9], the Long-tailed Skua feeds on berries, arthropods, small marine prey, or parasitizes other seabirds, but its breeding success is then greatly reduced, sometimes to nil [3], [8], [10]–[12]. Being the most pelagic of the three small *Stercorarius* species, little is known about its migration and the limits of individual wintering areas [3]. Recent studies based on satellite tracking described the initial post-breeding dispersal, but failed to document movements and distribution over the entire annual cycle [13].

As the Long-tailed Skua feeds to a large extent on lemmings, which are keystone prey over much of the Arctic, including in north-east Greenland [8], it has a major role in the dynamics of terrestrial vertebrate communities in the region. Indeed, when lemmings are abundant, Long-tailed Skuas are their most important predator (both quantitatively and qualitatively), killing more than one percent of the lemming population per day, which is more than the mean daily productivity of lemmings and hence leads to their gradual decline [8]. Understanding the non-breeding ecology of this major predator is therefore particularly important, firstly, to separate possible terrestrial (summer; Arctic) *versus* marine (winter; Atlantic) influences on population size and breeding success, particularly as the latter has declined recently in some key strongholds [14]–[15], and, secondly, because exchanges with the marine ecosystem can impact the functioning and dynamics of terrestrial communities [16]–[17]. Documenting the annual movements of the Long-tailed Skua and assessing the status of their flyways and staging areas, including levels of marine productivity, conservation issues and potential threats, and co-occurrence of other trans-equatorial seabirds, are the first steps of this quest.

By fitting geolocators (light-level loggers) to adult Long-tailed Skuas breeding in north-east Greenland and Svalbard (i.e. in populations relying on different food resources), the main aims of the present study were to (1) document the timing of migration and routes used by birds from these two representative populations in the north-east Atlantic region, (2) define the limits of individual wintering areas, (3) locate possible staging areas along the flyway, and (4) compare the non-breeding distribution of this species with that described recently for two other typical trans-equatorial migrants from the Arctic that are known to be kleptoparasitized by Long-tailed Skuas, Arctic Terns (*Sterna paradisaea*) and Sabine's Gulls (*Larus sabini*).

## Materials and Methods

### Ethics statement

Capture, handling and banding followed the North American Banding Council's code of ethics [18] and was approved by the ethical committee of the French Polar Institute, by the Government of Greenland (Ministry of Domestic Affairs Nature and Environment, Agency of fisheries hunting and agriculture; Permit Numbers 660113 and 647126), by the Norwegian Animal research Authority (Permit 2601) and by the Governor of Svalbard (Permits 00053-4, 00053-8).

Six adult Long-tailed Skuas were captured on their nests using remote-triggered nooses (on the nest) or a hand held netgun (off the nest) in Svalbard (Kongsfjorden, c. 79°N-12°E) in July 2010, and nine with bow nets in north-east Greenland (Zackenberg 74°29′N-20°35′W and Hochstetter Forland 75°09′N-19°40′W) in July 2010 and July 2011. All birds were measured, ringed, blood-sampled for DNA sexing (following [19]) and fitted with Mk18H geolocators (British Antarctic Survey, Cambridge, UK). The 1.9 g geolocators were attached to a drilled white plastic ring with a cable tie, and fitted to the tarsus. The entire package weighed c. 3 grams (equivalent to c. 1% of the average adult body weight).

All the birds that had been fitted with geolocators in 2010 (n = 8) and 2011 (n = 7) returned to their territories in the following year. Nine of these birds (six in Svalbard and three in Greenland) were recaptured after one year (Table 1). One of these birds had lost its logger, but the remaining eight loggers had recorded data for the previous 12 months, providing c. 3200 positions.

**Table 1:** Dates of capture, recapture and morphometrics of the nine Long-tailed Skuas fitted with geolocators in 2010–11 and recaptured in 2011–12.

Origin	Ring number (sex)	Dates (start/end)	Body mass (g)	Head (mm)	Wing (mm)	Tarsus (mm)	Tail (mm)
**Greenland**	5127016 (male)	15/07/2010	*n.d.*	*n.d.*	*n.d.*	*n.d.*	*n.d.*
		06/08/2011	272	*n.d.*	*n.d.*	*n.d.*	*n.d.*
	6238717 (male)	19/06/2011	263	70.7	322	35.8	*n.d.*
		12/06/2012	254	68.8	311	39.3	*n.d.*
	6238713 (male)	05/07/2011	282	74.6	328	43.9	*n.d.*
		15/06/2012	292	73.1	330	44.2	*n.d.*
**Svalbard**	6218052 (male)	05/07/2010	247	68.5	300	40.5	*n.d.*
		29/06/2011	246	68.4	286	41.7	286
	6218057 (male)	07/07/2010	227	73.3	307	41.3	*n.d.*
		02/07/2011	254	71.6	303	39.9	265
	6218053 (male)	05/07/2010	252	69.9	316	42.9	*n.d.*
		01/07/2012^a^	267	70.4	309	44	272
	6218051 (female)	04/07/2010	292	70.5	316	41.6	*n.d.*
		29/06/2011	283	70.0	319	41.7	301
	6218056 (female)	07/07/2010	220	67.8	302	40.5	*n.d.*
		02/07/2011	258	71.0	301	40.3	240
	6218059 (female)	10/07/2010	270	67.9	311	39.7	*n.d.*
		12/07/2011^b^	287	68.3	314	38.3	*n.d.*

a in order to give each bird the same weight in the analyses and figures, only positions collected between 5th of July 2010 and 1st of July 2011 were considered for this bird.

b this bird had lost the geolocator when recaptured.

### Data analyses

The Mk18H geolocators measure light intensity at 60 s intervals and record the maximum of these readings in every five minute interval. Following Frederiksen et al. [20], we first estimated positions (latitude from day and night length, and longitude from the time of local midday and midnight) using a range of sun elevation angles between −1.5° and −4.5°. We then empirically chose the best value obtained for each bird (−3° angle: n = 6; −2.5° angle: n = 2), according to how well the positions fitted to the shape of the continents. The geolocators provide two locations per day with an average error of <200 km [21], except during equinox periods and at high latitude in summer (i.e. in regions with 24 h daylight), when latitude and, in the latter case also longitude cannot be calculated. The two positions produced per day were averaged and, unless stated otherwise, the 3-day running mean of these daily averages (eqn 1) was used to estimate daily, weekly or monthly orthodromic (great-circle) distances and rates of travel (rather than flight speeds; see [22] and [23]).

We used the following equations, all in decimal degrees, to average daily positions 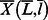 (eqn 1) and to calculate orthodromic distances (*D*_o_) between polar coordinates (eqn 2; adapted from [24]): 
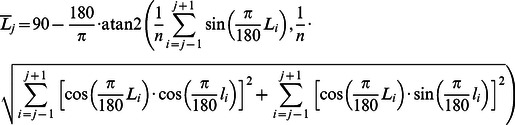
(eqn 1a)

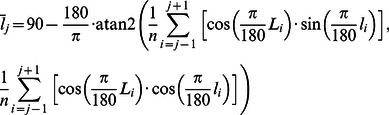
(eqn 1b)where 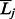 is the averaged latitude for day *j* (3-day running mean from *j−1* to *j+1*), 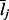 the averaged longitude, 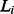
and 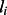 are respectively the latitude and longitude of daily positions *i*, and *n* is the number of polar coordinates (i.e. three in our case). Note that latitudes must be implemented negatively when south of the equator and longitudes negatively when west of the Greenwich meridian;
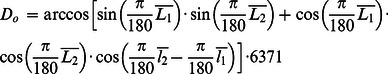
(eqn 2)where 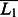
and 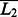
are respectively the latitudes (again in decimal degrees) of the two positions to be compared, 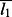
and 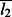
the longitudes of the same positions and 6371 the mean radius of the Earth in km. Note that the terms π/180 and 180/π have only been included here in order to present ready-to-use equations with positions in decimal degrees (i.e. when using angles in radians, these terms can be removed from eqns 1 and 2).

Missing legs of the migration routes (i.e. during equinox periods or in areas with 24 h daylight) were assumed to be linear or to follow flight paths that were parallel to the coast. Kernel density maps were produced in ArcGIS 9.3.1 software (ESRI Inc., Redlands, CA, USA) with a cell size of 20 km and a smoothing factor (search radius) of 200 km in order to be directly comparable to other recent studies [25]. A smoothing factor of 200 km also reflects the expected geolocation error [21], [26].

We define staging as any period of at least three consecutive days where distances between smoothed positions were smaller than 200 km. This approach is less conservative than the definition proposed by Warnock [27] and differs somewhat from some previous studies, but measuring changes in longitudes [28] or latitudes [25], [29] alone is not appropriate for species such as the Long-tailed Skua because: (1) this method would only properly discriminate between migration and staging periods on east-west or west-east flyways, and (2) since the distance between longitude lines declines toward the poles, this filter becomes more and more restrictive as birds move away from the equator, a major bias for Arctic breeding species.

## Results

### Timing of migration and flyways

**Figure 1:**
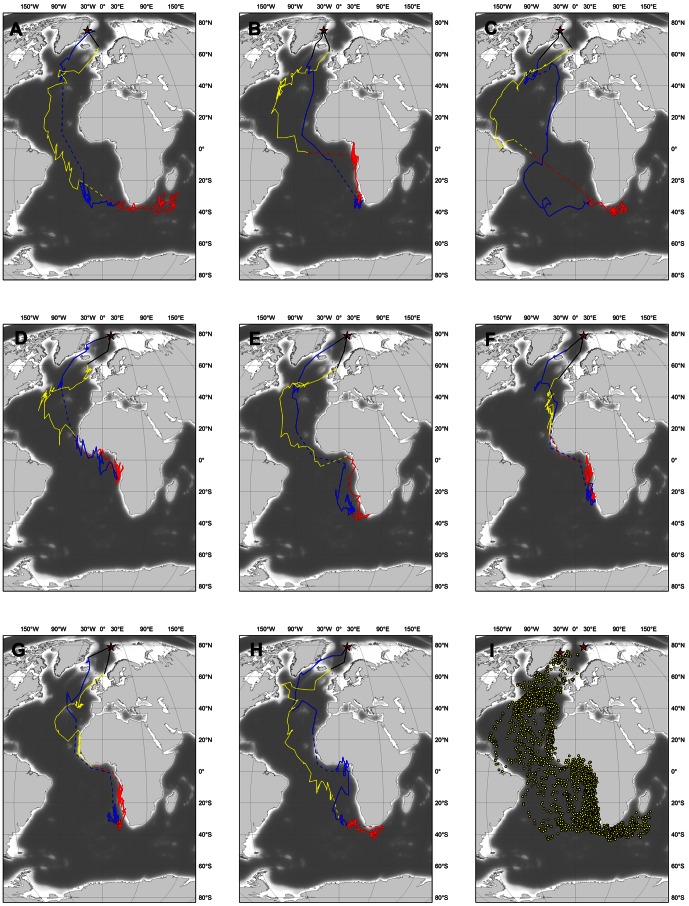
Flyways used by Long-tailed Skuas between their breeding grounds (red stars) and their wintering ground. (a–c) three males from north-east Greenland, (d–f) three males and (g–h) two females from Svalbard. Autumn movements (August–November) are in blue, winter movements (December–March) in red and spring movements (April–June) in yellow. Dashed lines represent interpolations (linear or parallel to the continents) for periods (equinox) when latitude could not be estimated. Black lines: same interpolations close to breeding grounds due to permanent daylight. The last panel (i) presents all raw positions (two per 24 h) obtained for these eight birds over one year.

**Figure 2:**
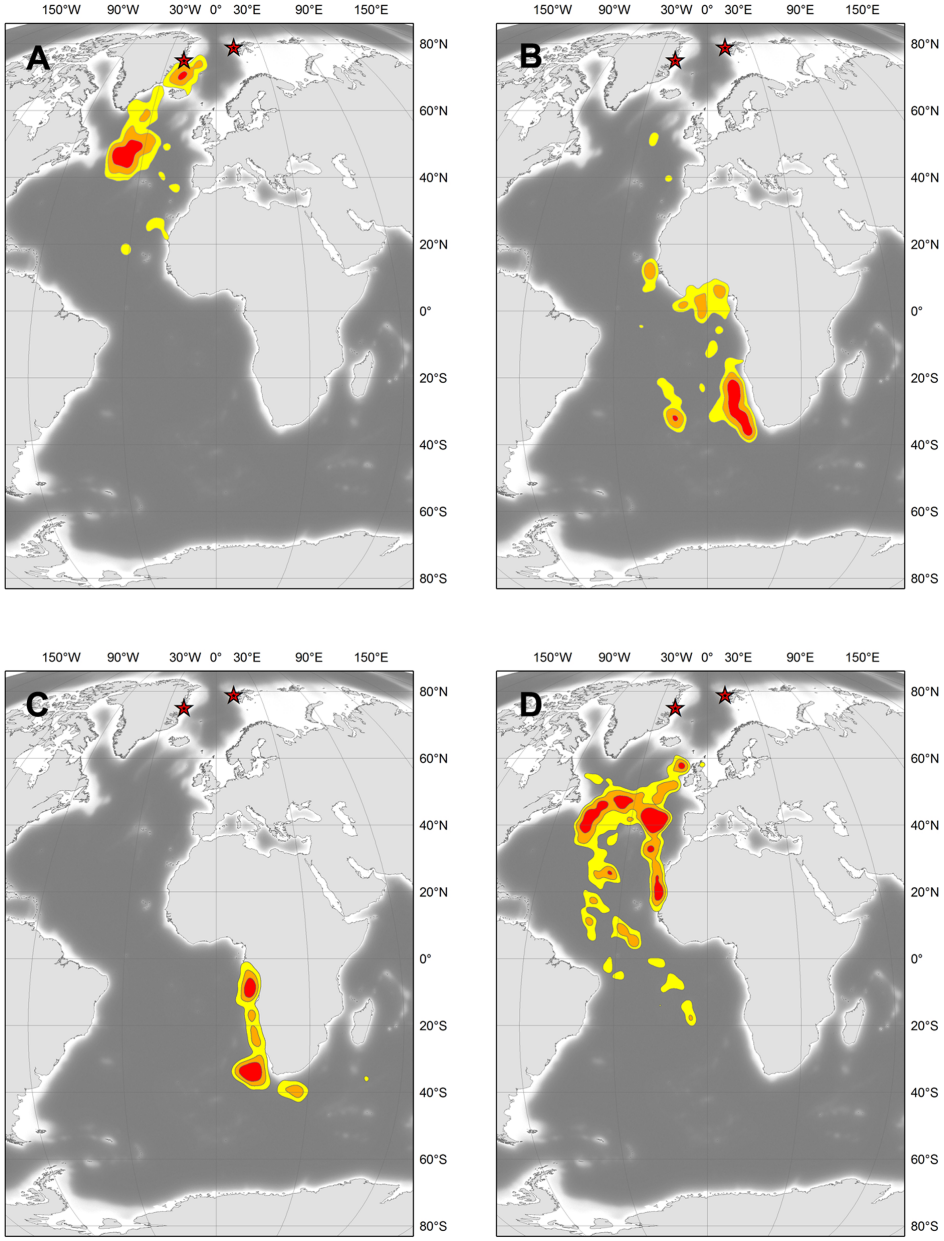
Kernel density distribution estimated for the Long-tailed Skuas. (a) from release to September 10^th^, (b) between October 10^th^ and November 31^st^, (c) December and January and (d) after April 10^th^. Contours represent densities of 25% (red), 50% (orange) and 75% (yellow).

All tracked birds started their post-breeding migration within a 10 d period during the second half of August (Table 2). All but one bird (which followed a route east of Iceland) first moved south-west along the Denmark Strait between Greenland and Iceland (Figure 1). After staging for some time in an area east of the Grand Banks of Newfoundland, Canada (Figure 2a), they continued towards the Cape Verde Islands and the west African coast (flying either east or west of the Azores), arriving by the beginning of September (latest arrival October 19; Table 2; see also [30]–[32]). From there the tracked birds continued southward, either on a coastal (inner Gulf of Guinea) or offshore route (see [33]), to their wintering grounds, where they arrived at some point during the long period between end September and late November (Figure 2b).

**Table 2:** Annual cycle based on the eight monitored Long-tailed Skuas (median values followed by ranges in brackets).

Start of the autumn migration^a^	21 Aug.	(14–24 Aug.)
Arrival in the West Africa/Cape Verde region	8 Sept.	(2 Sept–19 Oct.)
Arrival in the wintering area	24 Oct.	(26 Sept.–21 Nov.)
Start of the spring migration	21 March	(5 March–19 Apr.)
Earliest estimated arrival in Greenland^b^	28 May	(23 May–1 June)
Earliest estimated arrival in Svalbard^b^	4 June	(2–9 June)
Laying date in Greenland^c^	10 June	(9 June–9 July)
Laying date in Svalbard	17 June	(14–20 June)

a This represents the latest estimated start of migration for individuals. First geolocated positions were obtained when the birds were south of the constant daylight zone. We assumed that the birds started the migration one day before the first position was geolocated.

b Assuming direct flight along black lines as in Figure 1, from last geolocated position to the colony with the same speed as that of the average over the previous 10 days.

c Nest initiation dates were estimated by means of egg floatation [62].

The onset of the return, spring migration, also took place over a relatively long period, from March 5 to April 19 depending on the individual, but arrival on the breeding grounds was closely synchronized - always in early June, at most 2 weeks before all but one of these birds initiated laying (Table 2). On this northbound spring migration, all birds first used a more westerly and pelagic flyway than on the southbound autumn migration (Figure 1). Most birds returned to the same staging area they had used in the autumn (Figure 2d), but from there used a more easterly flyway than in August-September, returning to breeding grounds on a route between Iceland and Scotland (Figure 1).

Over their entire annual life cycle, Long-tailed Skuas mostly occupy or travel through the northern and south-eastern parts of the Atlantic Ocean (Figure 1i and 2). Only one bird approached close to the South American coast in autumn and two during their spring migration (Figure 1a and 1c). Three birds (including the latter two) rounded the Cape of Good Hope in winter, entering the south-west Indian Ocean, one travelling as far east as southern Madagascar, where Long-tailed Skuas have not been recorded previously [34].

### Wintering areas

Between December and March, all birds used wintering grounds located in the Southern Hemisphere between 0 and 40°S (Figure 1 and 2). During this period, five birds remained along the south-west coast of Africa between 0 and 15°E, between the Gulf of Guinea and the productive Benguela Upwelling (off the Namibian and South African coasts). Three birds (Figure 1: panels a, c and h) stayed further south and used more pelagic habitats in a narrower latitudinal (30–40°S) but wider longitudinal (0–50°E) area.

**Figure 3:**
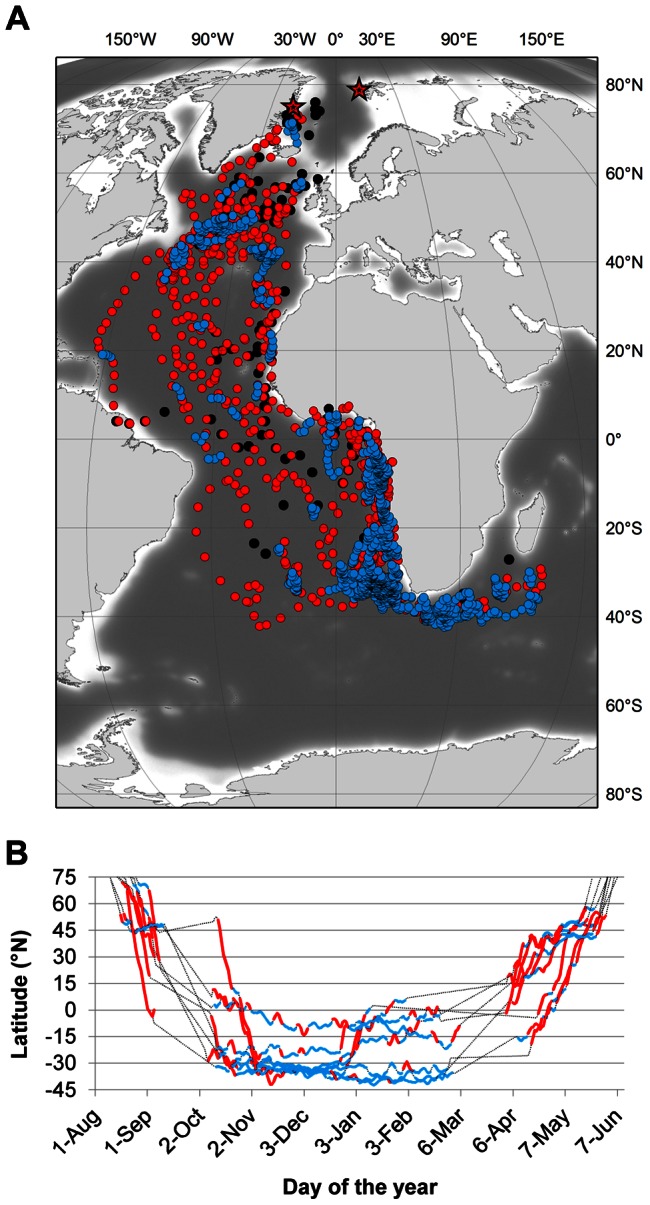
Staging areas (blue lines and symbols) used by Long-tailed Skuas. (a) geographical distribution of the staging areas used in the Atlantic and Indian Oceans; red colour is used for migration (i.e. “non-staging”) periods, blue colour for staging and black for periods when status could not be defined (i.e. when less than three successive days with daily positions). (b) individual differences in timing and latitude of staging.

The birds spent around five months on their wintering grounds, arriving in the second half of October, and began their return migration in March to early April (Figure 3b; exact departure dates are unknown in most cases because of proximity to the spring equinox; see Methods). The three birds that wintered offshore spent all of this period south of 30°S, whereas one bird never went further south than Angola and spent all the winter in the Gulf of Guinea north of 15°S (Figure 1d). The four other birds used two different wintering areas. They first went south to the Benguela upwelling but returned northward after two months (towards the end of December) and stayed offshore of the Angola and Gabon coasts (0–15°S) for the remaining 2–3 months (Figures 2c and 3a).

### Autumn and spring staging areas

Two staging areas were identified during the post-breeding migration (Figures 2a and 3). The first was in the Denmark Strait (68–71°N), where three birds from Svalbard spent up to one week shortly after their departure from breeding territories in August. The second and most important area was located off the Grand Banks of Newfoundland (Canada; c. 50°N). All the birds tracked in this study interrupted their migration when they reached the vicinity of this apparent hotspot (between 45 and 50°N) and staged for a few days to several weeks (maximum three weeks for one bird from Greenland).

Different routes and areas were used during the spring migration. Two of the birds that had spent the winter along the south-west African coast returned to Svalbard using an easterly and partly coastal flyway (Figure 1f and 1g), staging first along the Mauritanian Coast at c. 20°N and then following a route far off the Iberian Peninsula close to the Azores archipelago (c. 35–45°N; Figure 2d). These two birds respectively spent five and six weeks in these two staging areas (longest stay from 18 March to 23 May). All the other birds used a more westerly flyway, returning to the Grand Banks hotspot along an offshore route roughly parallel to the Mid-Atlantic Ridge (Fig 1a,b,c,d,e,h). Although the birds spent short periods (<1 week) in various staging areas during their return journey (in regions as distant as the Caribbean and north of Ireland; Figure 1cd and 3b), most staging periods were of >1 week and in areas between 30 and 50°N (Figure 3), within a crescent-shaped region centred around the Grand Banks hotspot (Figure 2d). Staging durations at this hotspot were 6, 17, 21 (2 birds) and 45 days (Figure 1h) and occurred between mid-April and late May (Figure 3b).

### Rates of travel

**Figure 4:**
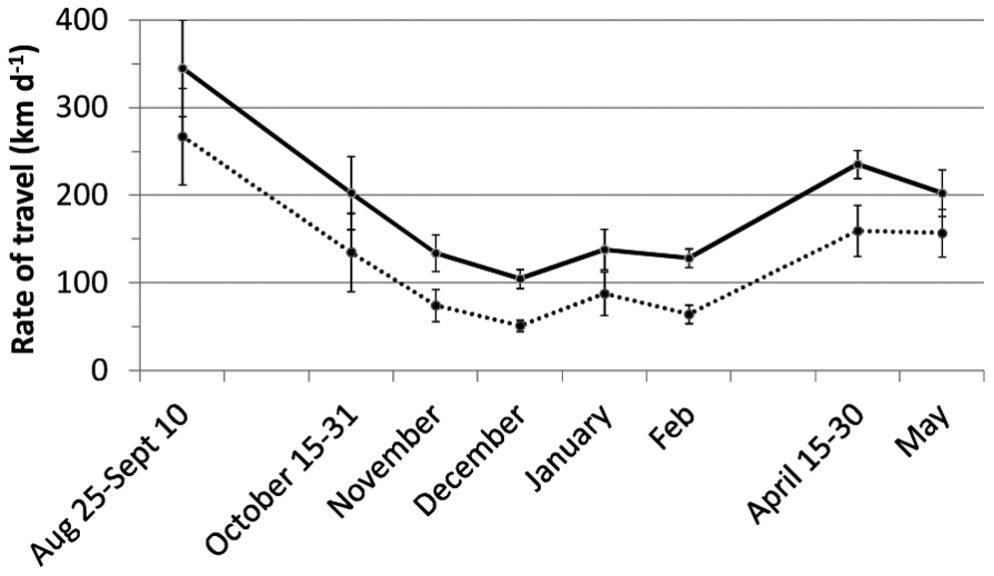
Daily (full line) and weekly (dotted line) rates of travel estimated for Long-tailed Skuas (in km d^−1^ ±S.E.).

Seasonal changes in travel rates (in km d^−1^) were calculated using daily and weekly positions (Figure 4). Daily rates of travel were greater in late August-early September, when birds moved on average 345 km d^−1^, than during the spring migration, when mean rates were only 235 (April 15–30) and 202 km d^−1^ (May). From November to February, when most birds were on their wintering grounds, daily rates of travel were two to three times lower than during migration (i.e. between 105 and 138 km d^−1^). These latter values are close to the error inherent in geolocator data [21], but the weekly rates of travel, mean values of which were always c. two to 10 times larger than the typical geolocation error of c. 200 km, present the same pattern of seasonal change. The difference between daily and weekly rates of travel was fairly constant over the year (mean: 62±11 km d^−1^; Figure 4).

### Total distance travelled

The total distance travelled over one year was on average 48,322 km and varied little between individuals (SD: 3549 km). Excluding distances travelled within the staging areas (including in winter), the estimated distance was 40,137 km. The straight-line distances between the breeding grounds and furthest point reached during the winter were 10,557–13,770 km (Table 3).

**Table 3:** Distances travelled by Long-tailed Skuas (Greenland and Svalbard birds combined; n = 8).

	Mean ± SD	Range
Maximum recorded distance from breeding ground	12 785±1007	10 557–13 770
Total distance travelled per year including staging periods	48 322±3549	43 909–54 162
Total distance travelled per year excluding staging periods	40 512±3249	36 961–46 015

## Discussion

Prior to this study, migration routes, staging sites and wintering areas of Long-tailed Skuas were poorly known, and based on at-sea observations. One exception is the recent satellite-tracking by Sittler et al. [13] of the post-breeding movements of four adults originating from different locations in north-east Greenland for 1.5–3 months until they reached the west African coast. As in Sittler et al. [13], all our birds staged in the “Grand Banks hotspot” (east of the Grand Banks and south of the Charlie–Gibbs fracture zone), an area apparently associated with the subpolar front [35] that has also been reported to be used by several other species of migrating Arctic seabirds [13], [20], [29], [35]–[38]. With the exception of the departure dates from this “Grand Banks hotspot”, which occurred earlier in the present study, migration patterns documented by Sittler et al [13] are in line with our own data, but we can now also provide information about the origin of birds using different wintering grounds. As they suggested, it appears that birds breeding in the north-east Atlantic region are using wintering areas along the coasts of Angola, Namibia and South Africa, where large numbers have already been reported by Lambert [39]–[40] and Ryan [41]. Some of these birds can probably be found further east (Figure 1a and 1c), as documented by Lambert [42], or as far west as the South American coast (Figure 1c). However, based on our data, the large number of birds reported by some authors to winter further south along the Falkland Current [43]–[44] and off the Chilean coast [45]–[48] are more likely to originate from different regions (e.g. Siberia and Alaska). Indeed, the only bird in our study that used a more westerly route close to South America did not remain in the Falkland Current and only passed through it during its post-breeding migration (not for staging or wintering; Figure 3a), most likely taking advantage of more favourable winds (see below).

### Individual variability

Although the main patterns described in this paper were common to all birds, we recorded some individual differences in timing, flyways, staging sites and wintering grounds. On their southbound migration, most birds remained in the east Atlantic Ocean, and only one made a long detour via the South American coast, possibly taking advantage of favourable counter clockwise winds in the Southern Hemisphere [49]. The track of this bird suggests the existence of a bi-directional post-breeding flyway similar to that described for the Arctic Tern [29]. More unexpected was the distinction on the wintering grounds between the three individuals that remained south of the Cape of Good Hope within a wide latitudinal but narrow longitudinal area, and the others that stayed along the south-west coast of Africa. During their spring migration, two birds of the latter group followed routes close to the west coast of Africa and Europe, whereas others used a more westerly flight path, returning to the staging hotspot off the Grand Banks of Newfoundland where all but one had staged in the previous autumn. None of the differences noted above were restricted to a given sex or geographical origin. Of the eight birds weighed in both years, two had lost weight (0.5–3.4%) and five had gained weight (3.5–15%) when recaptured (Table 1).

### Shared flyways and wintering grounds for trans-equatorial migrating Arctic seabirds

**Figure 5:**
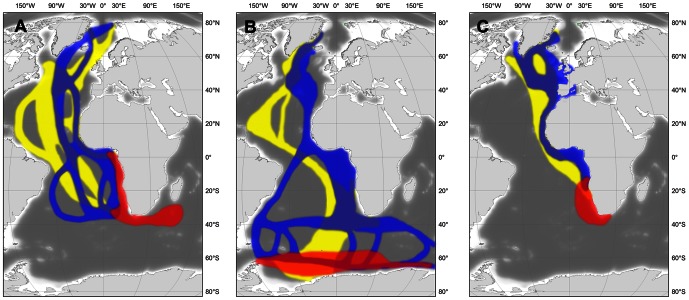
Major flyways and wintering grounds of the Long-tailed Skuas compared with two other sympatric seabirds. (a) Long-tailed Skua, (b) Arctic Tern and (c) Sabine's Gull. Contours are graphically inferred from Figure 1 (panels 1–8), Egevang et al [29] and Stenhouse et al [25], respectively. Blue: autumn flyways; Red: wintering grounds; Yellow: spring flyways.

Among the few tundra-nesting seabirds that are known to have a trans-equatorial migration, only the movements of Arctic Terns and Sabine's Gulls had been tracked previously in any detail [25], [29]. The new data we present for Long-tailed Skuas allow us to compare the migration patterns of these sympatric species (Figure 5), a comparison which is of particular interest since the Long-tailed Skua is a known to kleptoparasitize the two other species, both at the breeding and wintering grounds [3], [44]. The main conclusion is that regardless of the species, most of the birds monitored in the three studies headed towards the south-west coast of Africa during their post-breeding migration, after having staged (at least for Long-tailed Skua and Arctic Tern) in the Grand Banks hotspot and successively followed the coasts of West Africa and the Gulf of Guinea (Figure 5). During these transit periods, the Long-tailed Skua is the most pelagic migrant, with some birds (Figure 1b and 1e) staying far offshore (i.e. west of the Azores) until they cross the Equator. In contrast, many Sabine's Gulls follow the coasts of western Europe (with some even entering the Mediterranean Sea) and all follow the African coast off Morocco and south to their wintering areas (Figure 5c; [25]). The Arctic Tern is the only species that has a marked bi-directional flyway, with several birds using an alternative South American route (Figure 5b; [29]). In winter, Arctic Terns are only found in Antarctic waters, south of 60°S, while all Sabine's Gulls and most Long-tailed Skuas remain in the same region, off Namibia and South Africa. In spring, Arctic Tern and Sabine's Gull use different flyways until they reach the central North Atlantic at c. 50°N, whereas the Long-tailed Skua uses a wider flyway overlapping those of both the other species.

What is obvious from this comparison is that Arctic Tern and Sabine's Gull breeding in north-east Greenland, although both being tundra-nesting trans-equatorial migrants, only spend limited time in the same regions (i.e. on their partly overlapping post-breeding flyways) outside the breeding season (but Arctic Terns breeding in north-east Greenland can probably be found with Sabine's Gulls from other populations in spring; see e.g. [50]). In contrast, because Long-tailed Skuas use more extensive flyways, they are found in all but two of the regions used by the two other species during their annual cycles (i.e., coastal Europe visited by Sabine's Gull in autumn and Antarctic waters used by Arctic Tern in winter). This important overlap in flyways and wintering grounds raises the question of the functional relations that possibly exist between the Long-tailed Skua and these two parasitized species outside the breeding season. Although Long-tailed Skuas are known to kleptoparasitize many species in addition to the Arctic Tern and Sabine's Gull during winter [3], [40]–[41], our results show that they are always (i.e., year-round) found in regions that enable them to parasitize at least one of these two Arctic species. Similarly to their tight biogeographic link with lemming species (*Dicrostonyx* spp. and *Lemmus* spp.) in summer (with the exception of small populations in Svalbard and West Greenland) [10]–[11], Long-tailed Skua distribution outside the breeding season might, at least to some extent, be linked to the presence of its most familiar hosts, the Arctic Tern and the Sabine's Gull, with which it breeds in sympatry. Several offshore observations support this assumption, both in the north and south Atlantic (see e.g. [35], [40]–[41], [43]). The benefits of such a specialization could include, for instance easy matching of the annual cycle with the spatiotemporal distribution of these species, limiting the costs of seasonal behavioural adjustments to different kleptoparasitic hosts. Following Arctic Terns and Sabine's Gulls to the Southern Hemisphere also induces extra costs compared to the strategy used by most other Arctic seabirds, which winter in the Northern Hemisphere (including at least one other kleptoparasite [38]). However, given the mild climate and high productivity found in the south-east Atlantic region, the balance would probably remain largely positive for such an efficient long-distance migrant capable of travelling more than 500 km d^−1^ (Figure 4; see also Figure 2 in [13]).

### Conservation implications

Although the Long-tailed Skua is not currently threatened [51], its breeding success is highly dependent on the availability of Arctic rodents whose population dynamics are currently impacted by climate change in several Arctic regions [52]. For example, lemming predators, including Long-tailed Skuas, have declined recently in north-east Greenland where lemming populations have collapsed [14]–[15]. Breeding populations of Long-tailed Skua can tolerate such unfavourable periods for several years, mainly because the species is long-lived, site faithful, partly relies on energy reserves acquired in the marine environment to cover the costs of breeding (carry-over effect ; [53]) and its populations are thought to include large numbers of non-breeding floaters (Barraquand et al., unpublished work). However, if such lemming collapses become more frequent, last longer or extend geographically, then the size of some regional skua populations would be negatively impacted and their numbers on wintering grounds would also decline rapidly. In such a scenario, it would become increasingly important for the birds to find optimal feeding conditions on their staging and wintering grounds. The Grand Banks hotspot, Mauritanian coast and Azores must in this context be recognised as particularly important staging areas, while the African coast south of the Gulf of Guinea and the surroundings of the Cape of Good Hope (both eastwards and westwards), should probably be regarded as the main areas of conservation concern outside the breeding season. The last two regions are highly productive ecosystems, mainly due to the upwelling of cold waters (Benguela Current) and to a convergence zone (Agulhas Current). They host large numbers of seabirds, including both local breeding populations (e.g. the Vulnerable Cape Gannet *Morus capensis* and African Penguin *Spheniscus demersus*) and long-distance trans-equatorial migrants (the three Arctic species discussed above and others such as the Cory's Shearwater *Calonectris diomedea* [54]). In addition to the many direct anthropogenic threats faced by seabirds on these important staging and wintering grounds, rapid climate change (e.g. through trophic mismatch) represents a new and increasing challenge, and the demographic consequences of these interacting threats are difficult to foresee [2], [55]–[61]. This study underlines that the conservation of arctic breeding seabirds with trans-equatorial migrations depends on global ocean health and the conservation of important areas in both hemispheres.
